# On Carcinomas and Other Pathological Entities

**DOI:** 10.1002/cfg.497

**Published:** 2005

**Authors:** Barry Smith, Anand Kumar, Werner Ceusters, Cornelius Rosse

**Affiliations:** 1 Ifomis University of Saarbröcken Germany; 2 Department of Philosophy University at Buffalo and National Center for Biomedical Ontology 126 Park Hall Buffalo NY 14260 USA; 3 European Centre for Ontological Research University of Saarbröcken Germany; 4 Department of Biological Structure School of Medicine University of Washington Seattle WA USA

## Abstract

Tumours, abscesses, cysts, scars and fractures are familiar types of what we shall call *pathological continuant entities*. The instances of such types exist always in or on *anatomical structures*, which thereby become transformed into *pathological anatomical structures* of corresponding types: a fractured tibia, a blistered thumb, a carcinomatous colon. In previous work on biomedical ontologies we showed how the provision of formal definitions for relations such as *is_a, part_of* and *transformation_of* can facilitate the integration of such ontologies in ways which have the potential to support new kinds of automated reasoning. We here extend this approach to the treatment of pathologies, focusing especially on those pathological continuant entities which arise when organs become affected by carcinomas.


**Comparative and Functional Genomics**
*Comp Funct Genom* 2005; **6**: 379–387.
Published online in Wiley InterScience (www.interscience.wiley.com). **DOI:** 10.1002/cfg.497

**Research Article**

### On carcinomas and other pathological entities

Barry Smith[1,2,3]*, Anand Kumar[1], Werner Ceusters[3] and Cornelius Rosse[4]

[1] *Ifomis, University of Saarbrücken, Germany*
[2] *Department of Philosophy, University at Buffalo and National Center for Biomedical Ontology, USA*
[3] *European Centre for Ontological Research, University of Saarbrücken, Germany*
[4] *Department of Biological Structure, School of Medicine, University of Washington, Seattle, WA, USA*

**Correspondence to:*
*Barry Smith, Department of Philosophy, University at Buffalo, 126 Park Hall, Buffalo, NY 14260, USA.*
*E-mail: phismith@buffalo.edu*

Received: 24 May 2005
Revised: 1 October 2005
Accepted: 7 November 2005

### Abstract

**Tumours, abscesses, cysts, scars and fractures are familiar types of what we shall call *pathological continuant entities*. The instances of such types exist always in or on *anatomical structures*, which thereby become transformed into *pathological anatomical structures* of corresponding types: a fractured tibia, a blistered thumb, a carcinomatous colon. In previous work on biomedical ontologies we showed how the provision of formal definitions for relations such as *is_a*, *part_of* and *transformation_of* can facilitate the integration of such ontologies in ways which have the potential to support new kinds of automated reasoning. We here extend this approach to the treatment of pathologies, focusing especially on those pathological continuant entities which arise when organs become affected by carcinomas. Copyright © 2006 John Wiley & Sons, Ltd.**

**Keywords:** ontology; pathology; anatomy; cancer staging; carcinoma; pathogenesis

### Background

The Ontology of Biomedical Reality (OBR; Rosse *et al.*, 2005) provides a preliminary classification of organismal continuant entities, shown partially in Table 1.

Continuant entities are entities which endure self-identically through time while undergoing a variety of different sorts of changes of size, shape, location, internal structure and so forth (Grenon *et al.*, 2004). The OBR classification distinguishes two high-level universals in the realm of organismal continuants: *material anatomical entity* and *material pathological entity*, which are disjoint in the sense that they share no instances in reality.

In accordance with the classification schemes presupposed in standard treatises of pathology, OBR conceives the universal *material pathological entity* as comprehending subtypes such as *tumour, ulcer, portion of pus*, which have no equivalents in normal, healthy organisms.

In addition, however, we need to do justice to those anatomical structures which serve as the hosts or bearers of abnormalities of the types mentioned and which have, as a consequence, become predisposed to malfunction and disease. This means that in addition to universals such as *colon carcinoma* and *empyema of the lung*, which are instantiated by corresponding pathological lesions, we need also to include universals such as *carcinomatous colon* and *empyematous lung*, which are instantiated by those anatomical structures whose physiological functions have been altered by those lesions.

We thus modify the classification in Rosse *et al.* (2005) by recognizing two kinds of pathological continuant entity, which we shall call *pathological formation* and *pathological anatomical structure*, respectively. Instances of the latter serve as the bearers or hosts for instances of the former.

As in the original classification, so also here, we take the non-pathological universals from the Foundational Model of Anatomy (FMA; Rosse and Mejino, 2003; **http://sig.biostr.washington.edu/projects/fm/**) as our starting point. The FMA

Copyright © 2006 John Wiley & Sons, Ltd.

**Table 1.** A part of the OBR classification of continuant entities

| |
|---|
| 1. Material anatomical entity |
| (a) Anatomical structure |
| (i) Canonical anatomical structure |
| (ii) Variant anatomical structure |
| (b) Portion of canonical body substance (portion of urine, portion of blood) |
| 2. Material pathological entity |
| (a) Pathological structure (neoplasm, inflammatory structure, degenerated structure) |
| (b) Portion of pathological body substance (portion of pus, portion of amyloid) |

is a structured representation of the anatomy of instances (particulars, individuals), whose constituent nodes are representations of those 'multiply located anatomical entities (i.e. universals) that exist in the instances (particulars) that they subsume' (Rosse and Mejino, 2003).

The universal *anatomical structure* is defined by the FMA as follows:

> 'An anatomical structure is a material physical anatomical entity which has inherent 3D shape and is generated by coordinated expression of the organism's own structural genes.'

The particular entities which satisfy this definition, and which are thus instances of the corresponding universal, include cells and organs as well as cardinal body parts, such as the head and trunk.

For reasons outlined in Rosse and Mejino (2003), the FMA is restricted to anatomical entities which are 'typical' in the sense that they can be conceived as belonging to an 'idealized', healthy male or female adult human being (such entities are also identified in the literature of the FMA as 'canonical' entities). But there are also 'typical' entities in the realm of pathologies. The cases of small cell carcinoma of the lung and adenocarcinoma of the colon discussed in pathology textbooks are 'typical' in the sense that they possess the characteristics by which entities of the given types may be most readily distinguished from other pathological formations. It is the task of pathology as an empirical science to specify the characteristics by which subtypes and modifications of these 'typical' instances can be specified.

An anatomical structure is *pathological* whenever:

1. It has come into being as a result of changes in some pre-existing canonical anatomical structure through processes other than the expression of the normal complement of genes of an organism of the given type.
2. It is predisposed to have health-related consequences for the organism in question manifested by symptoms and signs.

An organism (or part of an organism) is *diseased* if and only if:

1. It includes among its parts pathological formations which:
2. Compromise the organism's physiological processes to the degree that they give rise to symptoms and signs.

Symptoms and signs, too, would require a detailed ontological treatment which, however, we do not attempt here.

An organism (or part of an organism) is *healthy* if and only if it is not diseased.

So long as a pathological continuant does not interfere with physiological processes, we have pathology but no disease. A pathological continuant entity can thus exist even in a healthy organism. A single transformed epithelial cell need give rise to no health-related consequences, but it is a cancer *in situ* at the cell level nonetheless.

In what follows, now, we stipulate that 'canonical' and 'variant' shall comprehend exclusively non-pathological instances of the corresponding anatomical universals. Pathological anatomical structures are thus distinguished from variant anatomical structures (such as *middle lobe of left lung*) by the fact that the latter are not predisposed to manifest health-related consequences.

### Varieties of pathological continuant

In the light of considerations discussed in the Background section, we enhance OBR by sorting pathological continuant entities into two ontologically disjoint categories, *pathological formation* and *pathological anatomical structure*. Following the scheme of OBR, we then distinguish in each category *independent* and *dependent* continuant entities. Independent continuant entities can be defined for present purposes as continuant entities which have mass (and are thus material); dependent

Copyright © 2006 John Wiley & Sons, Ltd. 

continuant entities as continuant entities which do not have mass (and are thus immaterial) (Rosse *et al.*, 2005).

Tumours and abscesses are examples of independent pathological continuants, and so also are carcinomatous colons, wounded knees, punctured eardrums and fractured tibias. Examples of dependent pathological continuants are wounds, punctures, fractures and abscess cavities. The latter belong ontologically in the same family as boundaries and holes (Casati and Varzi, 1994). Indeed, the relation between dependent and independent pathological continuants is formally analogous to the relation of boundary-dependence defined in Smith (1997).

Independent pathological continuants can now be subdivided into:

- Pathological formations, e.g. a carcinoma, a blister, an ulcer, which are newly formed continuant entities evolving in some larger anatomical structure.
- Pathological anatomical structures, e.g. a carcinomatous lung, a blistered thumb, an ulcerated colon.
- Portions of pathological body substance, e.g. a portion of pus, a portion of amyloid.

It is upon the first two of these categories that we shall concentrate here. Our task is to understand the relations between such continuant universals as *carcinomatous lung, lung* and *carcinoma.* We must first, however, touch briefly on pathological occurrent entities.

### Varieties of pathological occurrent

Like all organismal continuants, pathological continuant entities are tied in every case to *occurrent* entities (happenings, changes, events, processes), which unfold themselves through time in successive temporal phases (Rosse *et al.*, 2005; Grenon *et al.*, 2004).

As noted in Rosse and Mejino (2003), there are certain basic types of processes involving biological continuants which, in various combinations, bring about phenotypic changes on all levels of granularity. These are processes of *neogenesis, deletion* and *spatial or structural rearrangement of constituents*, the latter often manifested as processes of *invasion*.

Often these processes entail specific sorts of changes in a single anatomical structure which preserves its identity over time. An instance of a given type of canonical anatomical structure at one stage may be identical to an instance of a pathological anatomical structure at some later stage.

Following Smith *et al.* (2005; **http://obo.sourceforge.net/relationship/**), we call such processes *transformations*; they are types of phenotypic change which are observed not only in the aetiology of pathological continuants but also in embryonic development and in growth and ageing. A colon remains a colon, indeed it remains one and the same colon, even when some of its parts have been transformed into a tumour of a size capable of obstructing its lumen and disrupting the ordered arrangement of layers in the colon wall. An epithelial cell of the colon in which a carcinogenic transformation has taken place is one and the same entity as the canonical (healthy) colon epithelial cell which existed earlier.

In reflection of the existence of such transformations, the OBR classification has been revised in such a way as to include *pathological anatomical structure* as a subtype of *anatomical structure* (Figure 1).

Such transformations occur even in the case of congenital pathological continuants, where we can in every case identify embryonic or fetal canonical anatomical equivalents whose development into more mature forms has been arrested or interfered with as a consequence of the failure or disruption of developmental processes. Thus, in the case of congenital neoplasms, the lung is formed in an embryo of 4 or 5 weeks of gestational age and it has existed before any of its cells became neoplastically transformed. Similarly, various types of congenital cardiac abnormality correspond to embryonic or fetal canonical anatomical structures arrested at specific stages of cardiac development.

A second subfamily of phenotypic changes consists of processes of *derivation* (Smith *et al.*, 2005), where matter is reorganized in such a way as to give rise to new entities which take the place of entities existing earlier, as for example in cases of cell division or fusion. A process of neoplastic change may not alter the essential characteristics of the few epithelial cells it primarily affects (the cells retain their identity), but the tumour that results from the uncontrolled proliferation of these modified cells

Copyright © 2006 John Wiley & Sons, Ltd. 

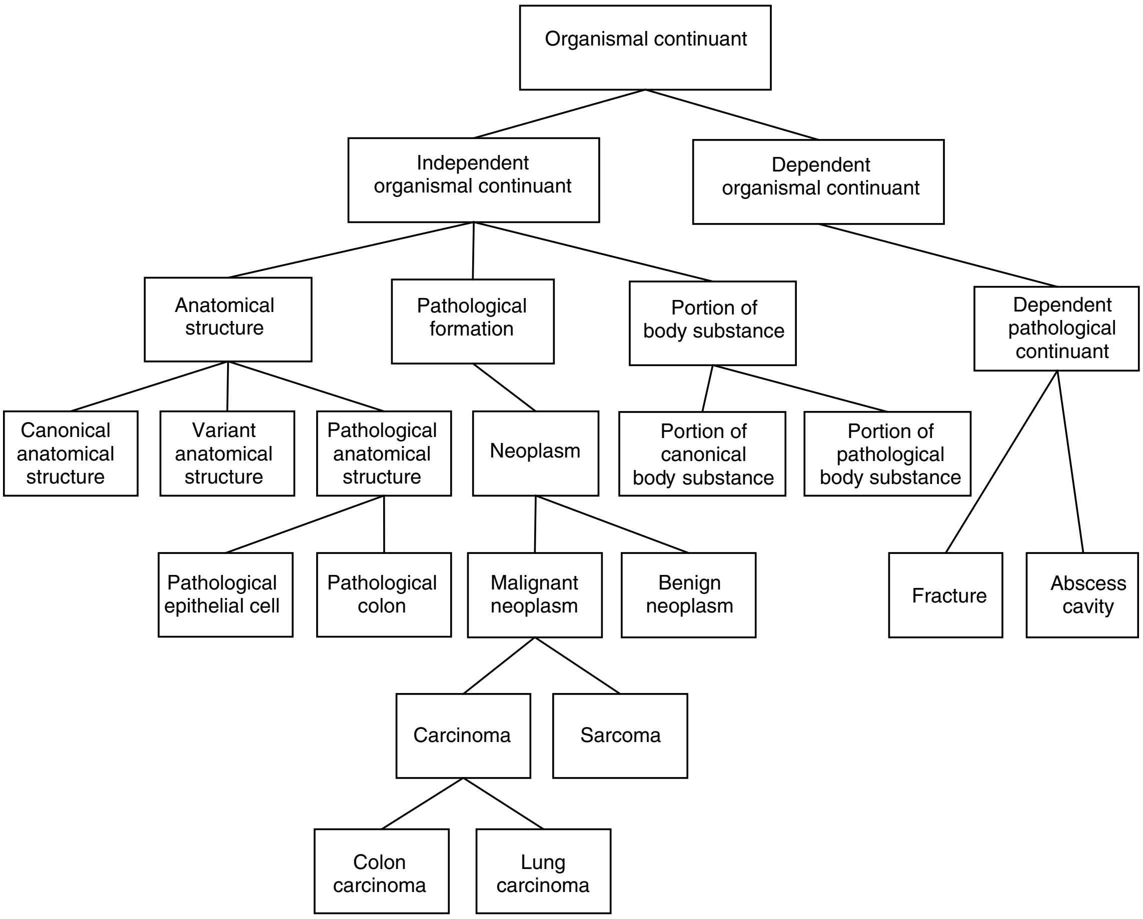

**Figure 1.** Revised OBR classification becomes a new entity in virtue of its phenotype. The tumour is *derived* from normal cells of the colon, but it is not a *transformation* of any pre-existing single entity.

Here again, such processes of derivation occur even in the case of congenital pathological continuants. Spina bifida arises through disruption of neural and vertebral fusion processes. The pathological continuants that we observe postnatally are then derived from abnormal embryonic or fetal structures, each of which in turn derives from a normal embryonic or fetal structure of an earlier developmental stage.

### Elements of a formal theory

Existing classifications of pathologies are contained, for example, in the International Classification of Diseases (ICD10; **http://www.cdc.gov/nchs/about/major/dvs/icd10des.htm**), SNOMED CT (**http://www.snomed.org/**), the NCI Thesaurus (**http://nciterms.nci.nih.gov/NCIBrowser**), the Pathology Descriptive Terminology (**http://cal.vet.upenn.edu/pathterm/menu.htm**) and OBO's Disease Ontology (**http://sourceforge.net/projects/diseaseontology/**). Unfortunately, none of these systems has the resources to support reasoning about pathologies in systematic ways. This is because none of them incorporates a formal ontological framework with the facility to represent the different types of pathological and non-pathological continuant entities and the relations between them.

In the classification summarized in Figure 1, the universal *anatomical structure* comprehends as subuniversals not merely *canonical* and *variant anatomical structure* but also *pathological anatomical structure*. Note that this is consistent with the

Copyright © 2006 John Wiley & Sons, Ltd.

definition of 'anatomical structure' provided above. (We here leave out of account discussions of pathological surfaces, pathological states and other non-material pathological continuant entities treated in Rosse *et al.* (2005), and also of biological pathologens such as bacteria and parasites, which are not parts of the organism in question. We also omit from the classification non-organismal substances, such as carcinogens, poisons and irritants of various sorts.)

Our classification can now be expanded through axioms asserting *is_ a* and *part_ of* relations between corresponding universals such as:

*canonical colonic epithelial cell is_a colonic epithelial cell*
*pathological colonic epithelial cell is_a colonic epithelial cell*
*pathological colonic epithelial cell is_a pathological anatomical structure*
*tuberculous lobe of left lung is_a pathological anatomical structure*
*canonical colonic epithelial cell is_a canonical structure*
*pathological colonic epithelial cell part_of colonic epithelium*

By making use of information in the FMA we can then infer for example that:

*pathological colonic epithelial cell part_of colonic mucosa*
*pathological colonic epithelial cell part_of colon wall*
*pathological colonic epithelial cell part_of colon*

and so on.

We use variables $A, B, C \ldots$ to range over universals (types) of continuants. We use $a, b, c, c'$, ... to range over the instances of such universals (particulars in reality, such as you and me, your tibia or your pleural cavity), and $t, t', \ldots$ to range over instants of time.

Following Smith *et al.* (2005), *is_a* and *part_of*, as relations between continuant universals, can be defined as follows:

*A is_a B* = def. for all $c$, $t$, if $c$ **instance_of** $A$ **at** $t$ then $c$ **instance_of** $B$ **at** $t$.
*A part_of B* = def. for all $a$, $t$, if $a$ **instance_of** $A$ **at** $t$ then there is some $b$ such that: $b$ **instance_of** $B$ **at** $t$ and $a$ **part_of** $b$ **at** $t$.

**Part_of**, here, is the instance-level part relation (which holds, for example, between this particular cell and this particular lung at this particular instant of time). This use of instance-level relations to define relations between universals, and also the all-some structure employed in the definition of *part_of*, are characteristic of almost all relations between universals of the sort treated by biomedical ontologies, although this fact is not always recognized consistently in such ontologies.

Note that it follows from our definition of *part_of* that *pathological colonic epithelial cell* stands in the *part_of* relation not only to *pathological colon* but also to *colon*.

Quantification over time in the above is designed to capture formally the temporal relations between instances of biological universals. Such relations have not been addressed in ontologies thus far, and even ontologies distinguishing successive stages of development of organisms or pathologies have not incorporated machinery for dealing directly with times (Aitken, in press). The reference to times allows us to do justice to the fact that one and the same entity can instantiate different universals and gain and lose parts in the course of time. Note that this reference is perfectly generic, which means that the definitions provided can be applied by users, even in the absence of specific time-indexed data.

### The genesis of pathological entities

Each pathological formation which is a carcinoma of the left lung stands in the instance-level **part_of** relation to that pathological left lung which serves as its host. On the level of universals, we have correspondingly:

*carcinoma of left lung part_of left lung*, although not, of course, the reciprocal relation (*left lung has_part carcinoma of left lung*).

The associated *transformation_of* relation is defined as follows (Smith *et al.*, 2005):

*A transformation_of B* = def. for all $t$ and all $c$, if $c$ **instance_of** $A$ **at** $t$, then there is an earlier time $t'$ at which $c$ **instance_of** $B$, and is illustrated for example by:

*red blood cell transformation_of reticulocyte*
*fetus transformation_of embryo*
*colon epithelial cell transformation_of colon epithelial cell precursor*.

Relations of this sort are not recorded even in an otherwise relation-rich terminology resource such

Copyright © 2006 John Wiley & Sons, Ltd. 

as the National Cancer Institute Thesaurus (NCIT), where, for example, no relations are asserted between the two classes *abnormal cell* and *normal cell*, not even that they have a common parent, *cell* (Ceusters *et al.*, in press). Transformation relations are also absent in the SNOMED CT terminology (**http://nciterms.nci.nih.gov/**).

A type of relation which we do find in SNOMED CT is that of location, which is there expressed for example in:

*lung cyst finding_site lung structure*

Better, however, would be to eliminate the epistemological connotations of '*finding_site*' by using a relation such as OBO's *located_in* (Smith *et al.*, 2005):

*A located_in B* = def. for all *a*, *t*, if *a* **instance_of** *A* **at** *t* there is some *b* such that: *b* **instance_of** *B* **at** *t* and *a* **located_in** *b* **at** *t*.

Here **located_in** is the location relation between instances obtaining for example between your brain and your cranial cavity at a given point of time. Significantly, *located_in*, the corresponding relation between universals, has the same all–some form which we encountered in the definitions of *part_of* and *transformation_of* above.

This framework can now be used as a platform for reasoning with axioms governing ontological relations in the domains of pathologies provided by other systems. *PathBase* (**http://eulep.anat.cam.ac.uk/Pathology_Ontology/MPATHdynamic.php**), for example, provides a subsumption hierarchy for pathological processes, to which are adjoined axioms pertaining to the corresponding pathological continuants, for example to the effect that:

*endoplastic reticulum defect is_a subcellular defect*

This axiom can be used with the colon cell assertions above to generate implications such as:

*pathological colon epithelial cell with endoplastic reticulum defect is_a pathological colon epithelial cell with subcellular defect*
*endoplastic reticulum defect located_in endoplastic reticulum*

and so on.

### Structures, patterns, processes and stages

The neoplastic processes involved in colon carcinoma are borne by an anatomical structure, the colon itself, as it is transformed over time. In their earlier stages these processes unfold themselves primarily in certain epithelial cells; in their later stages they will spread to the submucosa and muscle coats. Even as the latter become involved in and engulfed by the spreading cancer, however, they will remain unaffected so far as the nature of their cells is concerned, although the canonical arrangement of the components invaded by the cancer may be disrupted.

An inflammation and hypertrophy of the synovial membrane of the knee joint, similarly, is a pathological process which is initially confined to the synovial membrane itself, but then gives rise to degeneration of intra- and periarticular structures, as happens, for example, in the case of rheumatoid arthritis. Such multi-stage processes are captured in current bio-ontologies, for example, via the distinction between acute, subacute and chronic stages. In a complete representation one would need to specify the kinds of patterns associated with each such stage, and also the kinds of processes which yield them.

The processes with which we are dealing here are not processes of transformation but rather of invasion or infiltration, processes of a type which yield patterns of continuant entities related together in specific ways. Such patterns can be represented by means of compound terms, such as:

*muscle layer of colon invaded-by colon carcinoma*
*colon carcinoma of liver metastasis-of carcinoma of sigmoid colon*

and so on (Johansson, 1998). Here hyphens (-) are used in place of underscore separators (_) to mark the fact that we are dealing with *names* (of complex universals) rather than with *assertions* (of relations).

The instances of complex universals of the mentioned sorts are themselves complex continuant entities. We find in all anatomy-based classifications of carcinomas the generation of such complex names by means of syntactic operators of a type which have been recently investigated in relation to their use in the Gene Ontology (Ogren *et al.*, 2004; Kumar *et al.*, 2004; Mungall, 2004).

Copyright © 2006 John Wiley & Sons, Ltd. 

Some binary operators of this type have been used already in the above, for example, the operator 'with' in 'pathological colon epithelial cell with subcellular defect'.

As is shown in (Kumar and Smith, 2005), however, such operators have to be used with caution. The SNOMED term 'empyema of the gallbladder without mention of calculus' refers not to a special sort of empyema, but rather to a case of empyema that has been entered in a record in a certain way. Terms such as these can give rise to errors in reasoning (Kumar *et al.*, 2004). Moreover, because classifications developed with their aid must fall short of the ideal of single inheritance (in which every node has at most one *is_a* parent), these classifications themselves are subject to the characteristic kinds of errors which flow from *is_a* overloading (Kumar and Smith, 2005).

### Cancer staging

The framework sketched above can be exploited to capture, in a formal way, some of the information contained in systems for cancer staging, such as the TNM (tumour, node, metastasis) system, systems which do not, as currently constituted, sustain formal reasoning (American Joint Committee on Cancer, 2002). Here, 'T' refers to information about size and location pertaining to a primary tumour; 'N' records whether the cancer has metastasized to regional lymph nodes that drain fluid from the area of the tumour, and 'M' stands for metastasis, and indicates whether the cancer has metastasized to distant sites in the body, for example, from the colon to the liver.

A stage is conceived by the TNM system as a portion of the life or history of an entity, during which specific characteristics remain relatively constant. More correctly, however, it should be conceived as the pattern which endures — at a certain level of granularity — throughout the corresponding period, a pattern which can be captured formally by means of compound terms ('muscle layer of colon invaded-by colon carcinoma') capturing parthood, location and other relations between continuant entities along the lines indicated above.

The successive T stages of colorectal carcinoma are defined in the AJCC Cancer Staging Manual (American Joint Committee on Cancer, 2002) as follows:

Tis: Carcinoma *in situ*.
T1: Tumour invades submucosa.
T2: Tumour invades muscularis propria.
T3: Tumour invades through the muscularis propria into the subserosa, or into non-peritonealized pericolic or perirectal tissues.
T4: Tumour directly invades other organs or structures, and/or perforates visceral peritoneum.

Tis designates that stage during which cancer cells are confined to the luminal side of the epithelial basement membrane (intraepithelial) or the lamina propria (intramucosal), with no extension through the muscularis mucosae into the submucosa, the latter pattern being captured by the compound expression:

*colon submucosa invaded-by colon carcinoma*

T2 designates a stage in the unfolding of the carcinoma process during which the carcinoma has invaded the muscular layers of the colon wall.

N1 is a stage in which cancer has metastasized to one to four lymph nodes. M1 a stage where a metastasis is present in a part of the body not directly connected to the colon.

We can now assert, for example, that a pathological entity of the type:

*stage T2N1M1 colon carcinoma*

must be a transformation of either a T1N1M1 or a T2N0M1 carcinoma. We can infer further that, if a carcinoma is a transformation from T1N1M1 to T2N1M1, then a pattern of the type:

*muscularis mucosae invaded-by colon carcinoma*

has become instantiated.

If there is a transformation from T2N0M1 to T2N1M1, then the last process to take place before this transformation was of the type *lymph node involvement*. If there is a transformation from T2N1M0 to T2N1M1, then the last process to take place was of the type *metastasis to distant site*. And so on.

### Classifying carcinomas through the FMA

To create a robust classification of carcinomas, we need to find ways of linking the nodes of an

Copyright © 2006 John Wiley & Sons, Ltd. 

ontology of pathological continuant entities with appropriate nodes in the FMA and in reference ontologies of attributes, of diseases, of molecular biology, and so forth. The corresponding relations will be either synchronic (*is_a, part_of, located_at*, etc.) or diachronic (*derived_from, transformation_of*).

The FMA, as already noted, does not take account of pathological continuant entities within its hierarchy of anatomical universals. On the basis of axioms of the sorts presented above, however, we can use the FMA as a valuable resource to support reasoning about carcinomas.

The goal is to realize a scenario in which each given type of (for example) Tis small cell carcinoma would be represented as a node in a reference ontology of pathological continuant entities, and linked via the *located_at* relation to the FMA and to a cancer staging knowledge base in a way which would allow us to infer, for example, that the carcinoma in question is located in the mucosa of a respiratory bronchiole in the lateral basal segment of the left lung. Compound expressions, such as 'carcinomatous mucosa of a respiratory bronchiole in the lateral basal segment of the left lung', should not then be used to refer to universals in a pre-existing reference ontology. Rather, they should be generated on the fly to meet the specific needs of the reasoner in specific types of contexts.

If we generate a strictly location-based classification of carcinomas, via pointers going from the FMA to a pathology reference ontology, then the classification thereby generated would have the advantage that it would be more complete than a post-coordinated ontology of the type that is currently available in terminologies and ontologies such as SNOMED CT or the NCI Thesaurus, as it will necessarily take care, in automatic fashion, even of rare carcinomas. Thus, the universal *carcinoma of wall of alveolar duct* has instances in physical reality, but they are encountered too infrequently to be included in the usual disease ontologies.

Unfortunately, however, it would be too daunting a task to generate a new ontology reflecting all the different ways in which anatomical continuants may become tainted by the presence of pathological continuants of different sorts. For such an ontology would need to duplicate essentially the entire FMA for every kind of pathology universal, thus not only for *cancerous colon* and *carcinomatous colon*, but also for *sarcomatous colon, inflamed colon, acutely inflamed colon, chronically inflamed colon, atrophied colon, hypertrophied colon, colon containing parasite, colon containing parasite of type A, colon containing parasite of type B*, and so on.

We should not, therefore, strive to create a reference ontology along all the axes that prevail in current terminologies, but rather build a reference ontology of types of pathologies which can be used, together with the FMA and other domain reference ontologies, to generate local classifications according to specific needs. This would bring also the advantage that we can preserve the benefits of single inheritance in reference ontologies, even if we need to accept multiple inheritance in classifications created for specific purposes.

### Reasoning with the FMA

Given a classification of types of carcinomas based on the anatomical ontology of the FMA along the lines described, we could use the *is_a* and *part_of* relations present in the FMA to derive relations between the corresponding carcinoma structures on the basis of rules, such as:

from: *A is_a B* (in FMA)
infer: *carcinoma of A is_a carcinoma of B*

yielding, for example:

*carcinoma of lung is_a carcinoma of organ*

Of course, we also have:

*carcinoma of lung is_a carcinoma of anatomical entity*

and while the latter assertion captures no knowledge which is of immediate clinical significance, it may be of importance in ensuring completeness of the set of inferences we can make in a reasoning system.

We also have the rule:

from: *A part_of B* (in FMA)
infer: *carcinoma of A is_a carcinoma of B.*

Thus, from:

*ascending colon part_of colon*

Copyright © 2006 John Wiley & Sons, Ltd.

we can infer:

*ascending colon carcinoma is_a colon carcinoma*

And from:

*upper lobe of left lung part_of left lung*

we can infer:

*carcinoma of upper lobe of left lung is_a lung carcinoma*

A special issue arises where we employ anatomical expressions containing modifiers like 'whole' or 'complete'. The *part_of* components of the organ *left lung* include *upper lobe of left lung* and *lower lobe of left lung*. These form an exhaustive partition, so that the mereological sum of the two organ components is the whole left lung. While within the FMA '*whole left lung*' and '*left lung*' are treated as synonyms, for purposes of the classification of disorders the two expressions need to be distinguished (Hahn *et al.*, 2004). This is because we have, for example:

*upper lobe of left lung part_of whole left lung*

*lower lobe of left lung part_of whole left lung*

*carcinoma of upper lobe of left lung is_a carcinoma of left lung*

but not:

*carcinoma of upper lobe of left lung is_a carcinoma of whole left lung.*

### Conclusion

We have sketched a formal approach to the ontology of pathological continuant entities, resting on the distinction between two types of pathological continuant entity, called *pathological formations* and *pathological anatomical structures*, respectively. The framework is intended to support new types of reasoning about pathological entities and about the ways in which they develop through time, for example, in the domain of cancer staging.

### Acknowledgements

This paper was written under the auspices of the Wolfgang Paul Program of the Alexander von Humboldt Foundation, the European Union Network of Excellence on Medical Informatics and Semantic Data Mining, the Volkswagen Foundation under the auspices of the project 'Forms of Life', and the National Center for Biomedical Ontology.

### References

Aitken S. 2005. Formalizing concepts of species, sex and developmental stage in anatomical ontologies, *Bioinformatics* **21**(11): 2773–2779.

American Joint Committee on Cancer. 2002. *AJCC Cancer Staging Manual*, 6th edn. Springer; New York, NY: 113–124.

Bodenreider O, Smith B, Burgun A. 2004. The ontology–epistemology divide: a case study in medical terminology. *Formal Ontology and Information Systems (FOIS) 2004*. IOS Press: Amsterdam; 185–195.

Casati R, Varzi A. 1994. *Holes and other Superficiliaties*. MIT Press: Cambridge, MA.

Ceusters W, Smith B, Goldberg L. 2005. A terminological and ontological analysis of the *NCI Thesaurus*. *Methods Informat Med* **44**: 498–507.

Grenon P, Smith B, Goldberg L. 2004. Biodynamic ontology: Applying BFO in the biomedical domain. In *Ontologies in Medicine*, Pisanelli DM (ed.). IOS Press: Amsterdam; 20–38.

Hahn U, Schulz S, Kornél G. 2004. Mereological semantics for bio-ontologies. Proceedings of Twentieth National Conference on Artificial Intelligence (AAAI 2004) 257–262.

Johansson I. 1998. Pattern as an ontological category. *Formal Ontology and Information Systems (FOIS)*. IOS Press: Amsterdam; 86–94.

Kumar A, Smith B, Borgelt C. 2004. Dependence relationships between Gene Ontology terms based on TIGR gene product annotations. Proceedings of CompuTerm 2004: 3rd International Workshop on Computational Terminology, Geneva, 2004, 31–38.

Kumar A, Smith B. 2005. Oncology Ontology in the NCI Thesaurus. *Artificial Intelligence in Medicine Europe (AIME) 2005*. (Lecture Notes in Computer Science 3581), 213–220.

Mungall C. 2004. OBOL: integrating language and meaning in bio-ontologies. *Comp Funct Genom* **5**: 509–520.

Ogren PV, Cohen KB, Acquaah-Mensah GK, Eberlein J, Hunter L. 2004. The compositional structure of Gene Ontology terms. *Pacific Sympos Biocomput* **9**: 214–225.

Rosse C, Kumar A, Mejino JLV, *et al.* 2005. A strategy for improving and integrating biomedical ontologies. Proceedings of the AMIA Symposium, Washington, DC. 639–643.

Rosse C, Mejino JLV Jr. 2003. A reference ontology for bioinformatics: the Foundational Model of Anatomy. *J Biomed Informat* **36**: 478–500.

Smith B, Ceusters W, Klagges BER, *et al.* 2005. Relations in biomedical ontologies. *Genome Biol* **6**: R46.

Smith B. 1997. On substances, accidents and universals. In defence of a constituent ontology. *Philos Papers* **27**: 105–127.

Copyright © 2006 John Wiley & Sons, Ltd. 
